# Evaluation of the Usefulness of Sorbents in the Remediation of Soil Exposed to the Pressure of Cadmium and Cobalt

**DOI:** 10.3390/ma15165738

**Published:** 2022-08-19

**Authors:** Jadwiga Wyszkowska, Agata Borowik, Magdalena Zaborowska, Jan Kucharski

**Affiliations:** Department of Soil Science and Microbiology, University of Warmia and Mazury in Olsztyn, Plac Łódzki 3, 10-727 Olsztyn, Poland

**Keywords:** cadmium, cobalt, soil microbiome, soil enzymes, sorbents, *Helianthus annuus* L.

## Abstract

An undesirable side effect of economic progress is increasingly severe pollution with heavy metals, responsible for the degradation of ecosystems, including soil resources. Hence, this research focused on examining six adsorbents in order to distinguish a reactive mineral with the highest capacity to remediate soils contaminated with heavy metals. To this end, the soil was polluted with Co^2+^ and Cd^2+^ by applying the metals in concentrations of 100 mg kg^−1^ d.m. The extent of soil equilibrium disturbances was assessed by evaluating the response of the soil microbiome, activity of seven soil enzymes, and the yields of *Helianthus annuus* L. Six sorbents were evaluated: a molecular sieve, expanded clay (ExClay), halloysite, zeolite, sepiolite and biochar. Co^2+^ and Cd^2+^ proved to be significant inhibitors of the soil’s microbiological and biochemical parameters. Organotrophic bacteria among the analysed groups of microorganisms and dehydrogenases among the soil enzymes were most sensitive to the effects of the metals. Both metals significantly distorted the growth and development of sunflower, with Co^2+^ having a stronger adverse impact on the synthesis of chlorophyll. The molecular sieve and biochar were the sorbents that stimulated the multiplication of microorganisms and enzymatic activity in the contaminated soil. The activity of enzymes was also stimulated significantly by zeolite and sepiolite, while the growth of *Helianthus annuus* L. biomass was stimulated by the molecular sieve, which can all be considered the most useful reactive materials in the remediation of soils exposed to Co^2+^ and Cd^2+^.

## 1. Introduction

Soil is a natural resource with an immeasurable value, albeit it is also finite and non-renewable [1]. Rapid and uncontrollable urbanisation and industrialisation threaten the quality of soil and its long-term use, leading to the loss of its priority functions [2]. Excessive exploitation of soil by unsustainable agriculture inducing global-scale soil degradation is progressing at an alarming pace. Every year, around 10 million ha of arable land is considered to be unproductive [3], and the total growing area of degraded soils is now estimated at over 24% of the world’s soils [2]. The key challenge is, therefore, to increase or maintain crop yields without causing further degradation of the Earth’s ecosystems, in particular soils [4]. The challenge is further exacerbated by the growing demand for agricultural products for the rapidly growing human population in the world, predicted to reach 8.9 billion people in 2050 [5].

The problem of agricultural soil contamination with heavy metals calls for drastic and effective solutions [6]. Concentrations of these heavy metals surpassing the threshold values have negative effects on both the fertility of soils and on the growth, photosynthesis and productivity of crops, which in consequence, affects the health of humans and animals [7]. Apart from Cr, Ni, Cu, Zn, Pb, Hg and As, the list of most toxic heavy metals includes cadmium and cobalt [8,9].

Contamination with cadmium has grown to be a major global problem as the allowable threshold amounts of this xenobiotic in crops are exceeded [10]. Worldwide, the average content of cadmium in the soil is in the range of 0.01–1 mg kg^−1^ d.m. of soil [11], while its average content in soil exposed to an excess concentration of this metal can be as high as 20–800 mg kg^−1^ d.m. of soil [12]. In 2020, cadmium was added to the list of CRM (Critical Raw Materials) candidates [13]. This heavy metal penetrates soils mainly from anthropogenic sources, such as smelting and refining copper and nickel, combustion of fossil fuels, application of phosphorus fertilisers [14] as well as from landfill leachate, agricultural land and mining waste, mostly from zinc and lead mines [15]. Nowadays, cadmium is needed for the manufacture of Ni-Cd batteries, pigments and PVC [16,17]. In turn, cobalt draws attention, having been classified by the Agency for Toxic Substances and Disease Registration (Atlanta, GA, USA) as the 52nd of 275 substances defined as priority ones [18]. A large share of the global cobalt production output is associated with the extraction of such raw materials as copper (55%) and nickel (35%). Arsenic extraction also plays a significant role in the production of this heavy metal [19,20]. With a wide range of technologically advanced applications of cobalt in catalysers, batteries, magnets or high-strength materials, the global production index of this metal is now the highest ever, at 140 kt (kilotons) a year [21]. The biggest producers of refined cobalt are China (67%), Finland (11%), and Canada (5%), where the market is dominated by the batteries industry, responsible for 58% of cobalt consumption [19,22].

Undoubtedly, problems caused by soil contamination with heavy metals can be minimised by using methods based on precipitation and ultrafiltration. However, these processes are expensive and ineffective at low concentrations of heavy metals. In turn, adsorption is distinguished by its high potential for elimination, recovery and recycling of heavy metals [23]. At present, research is gaining attention on high integrity minerals used as sorbents of heavy metals and on biochar manufactured from organic raw materials submitted to pyrolysis. Biosorbents change the soil’s pH and promote the sorption of this pool of heavy metals on their surface or induce encapsulation of heavy metals in their crystalline structure [24,25,26,27].

Remediation of soils polluted with heavy metals is imperative. Studies on heavy metals, including cadmium and cobalt, have revealed correlations between these metals and a higher incidence of lung cancer and gastric cancer [28] as well as neurological and metabolic disorders [29]. On the other hand, cobalt, as an active component of cobalamin (vitamin B_12_), participates in the catalysis of the regeneration of erythrocytes and is engaged in the proper functioning of the brain and nervous system [30]. Mechanisms involved in the activity of heavy metals are similar in biotas on all trophic levels because they predominantly act on the cellular level in highly conservative systems and are typically cytotoxic and genotoxic [31]. They mainly induce the peroxidation of lipids and changes in the cell membrane. They also bind thiol groups of proteins, leading to the loss of their functionality [32]. Metal cations inhibit transcription and replication of the DNA, as well as alter the DNA’s structure [33].

The fate of cadmium and cobalt, most often present in the soil solution as cations Cd^2+^ and Co^2+^, is moderated by precipitation, mineral dissolution, ion exchange, adsorption, desorption and the uptake by plants [34]. Both cadmium and cobalt retard the growth of plants. They are also responsible for oxidative stress and depressed uptake of essential nutrients, as well as a deficit of photosynthetic pigments and chlorosis of leaves [35,36]. However, hyperaccumulator plants are able to survive and grow in the presence of high levels of heavy metals and can accumulate up to 100 µg of metal g^−1^ of the plant [37]. Then, the absorption of metals is regulated by the response of molecular pathways. This process occurs both inter- and extracellularly on the symplastic and apoplastic routes, respectively [38].

Owing to the chemical dialogue based on interactions between plants and the microbiome of their roots, stimulated by metallophores and other chelating agents, plants are capable of responding to the stress induced by heavy metals. Metallophores, secondary metabolites of plants and bacteria, bind heavy metals, including cadmium, or else enhance their mobilisation, thereby stimulating phytoextraction [39,40]. Furthermore, metallothioneins, rich in cysteine with four Zn^2+^ or Cd^2+^ binding sites, are engaged in the detoxication and storage of heavy metals. Cadmium is a metal that induces the synthesis of metallothioneins [41]. Microorganisms transform heavy metals to less toxic forms by methylation or redox reactions, which alter the solubility of heavy metals in soil [1,40]. During the process of biosorption, metal cations attach to the negatively charged cell surfaces and release exopolysaccharides (EPS) from cells, which stimulate the non-specific binding of metals [42]. It is also worth underlining that the pollution of soil with heavy metals creates a selective pressure, promoting species of bacteria that are resistant to this pool of heavy metals [43]. Because studies on changes in the quality of soil induced by heavy metals must be based on properties that respond rapidly to changes in the environmental stress, the biological indicator most often recommended, other than the response of microbiomes, is the activity of soil enzymes [44,45]. Heavy metals may produce an inhibitory effect on soil enzymes by interacting with the active sites of enzymes, complexes of substrates and by denaturation of proteins of these enzymes [46].

Two facts, namely the sunflower (*Helianthus annunus* L.) showing high tolerance to heavy metals [47] and the use of a wide range of sorbents for remediation of soils [23], gave rise to the imperative to conduct comprehensive studies in order to determine and compare the sensitivity of microorganisms and soil enzymes to the pressure of cadmium and cobalt in soil cropped with sunflower and submitted to remediation with the application of a molecular sieve, halloysite, sepiolite, expanded clay and biochar.

## 2. Materials and Methods

### 2.1. Soil

The type of soil used in the experiment is common in the north-eastern part of Poland. According to the FAO taxonomy [48], it is a Eutric Cambisol. The soil in a controlled plant-growing experiment was taken from the arable humic horizon, from a depth of 0–20 cm, at the Experimental Station in Tomaszkowo (NE, Poland, 53.713° N, 20.432° E). It was then transported to the Teaching and Experimental Centre of the University of Warmia and Mazury in Olsztyn (NE, Poland, 53.759° N, 20.453° E). In terms of the grain-size composition, it was sandy loam, containing 60.63% of sand (fractions 0.05–2.0 mm), 35.99% of silt (fractions 0.05–0.002 mm) and 3.38% of clay (fraction < 0.002 mm). The basic properties of the soil are presented in Table 1.

### 2.2. Procedure for Setting Up and Conducting the Experiment

Pot trials were conducted in a greenhouse in polyethene pots with a capacity of 3.0 dm^3^. The yearly average air temperature in the months when the experiment was carried out (June–July) in NE Poland was 17.7 °C, and the average rainfall was ca 83.5 mm. The experiment was conducted in three series with three replicates. The first series was composed of non-polluted soil, and the second one was set up on non-polluted soil with the sorbents, i.e., a molecular sieve, halloysite, sepiolite, expanded clay, biochar and zeolite Bio.Zeo.S.01, the third series consisted of soils polluted with cadmium and cobalt and four series were composed of polluted soil with cadmium and cobalt treated with the sorbents, i.e., a molecular sieve, halloysite, sepiolite, expanded clay, biochar and zeolite Bio.Zeo.S.01. All sorbents were applied in doses of 10 g kg^−1^ d.m. of soil. The heavy metals were applied in amounts of 0 and 100 mg kg^−1^ d.m. of soil. Cadmium was applied as 3CdSO_4_·8H_2_O, and cobalt as CoSO_4_·7H_2_O. Prior to packing soil, previously sifted through a sieve (5 mm mesh) to polyethene pots, each batch was mixed thoroughly with the sorbents, heavy metals and fertilisers, according to the experiment’s design. The fertilisers N, P, K and Mg were applied in all series of the experiment once before sowing the crops. The soil was enriched with 110 mg N as CO(NH_2_)_2_, 45 mg P as KH_2_PO_4_, 110 mg K as KH_2_PO_4_ and KCl, and 20 mg Mg as MgSO_4_·7H_2_O. Afterwards, the soil moisture content was raised to 60% by adding distilled water. The test plant was the sunflower *Helianthus annuus* L. The emergence of *Helianthus annuus* L. took place 5 days after sowing. The earliest pathological symptoms (yellowing) in the pots with cobalt were observed after 12 days. The sunflower’s greenness index (SPAD) was determined at the stage of six leaves unfolded, that is, BBCH (Biologische Bundesanstalt, Bundessortenamt and Chemical Scale) 16, based on the content of chlorophyll determined with a MINOLTA SPAD 502Plus (KONICA MINOLTA, Langenhagen, Germany). The harvest of the sunflower’s aboveground parts and roots was performed at stage BBCH 35.

### 2.3. Characteristics of Sorbents

A molecular sieve (Grace Davison Company, Columbia, New York, NY, USA) is hydrated aluminosilicate with micropores of a diameter less than 0.3 nm. This is a product sold under the trade name Silosiv A3. It is a zeolite with a three-dimensional pore system with pH*_KCl_*—8.5. Halloysite (Halosorb minerals sorbents, Intermark, Gliwice, Poland) is a silicate mineral (kaolinite group). This mineral is classified as a representative of clay minerals, Al_2_Si_2_O_5_(OH_4_). It is the weathering product of the Tertiary basaltic rock. The pore diameter is 10–20 nm [62]. Sepiolite (Sepiolsa Minersa Group, Getxo, Spain) is a naturally occurring clay mineral (Mg_4_[Si_6_O_15_(OH)_2_]·6H_2_O). It has a fibrous texture with a pore diameter of 1.4 nm [63,64]. Biochar (Fluid, Sędziszów, Poland) is produced from organic waste, such as wood production waste in sawmills, energy willow and miscanthus. It possesses a well-developed internal network of pores with a diameter of 5 to 50 nm [65]. Expanded clay (GardenGURU, Piła, Poland) is a light aggregate of grains 1.6–8 mm in size. It is burnt from loamy clay at 1150–1200 °C. Zeolite Zeolit Bio.Zeo.S.01 (BioDrain, Rzeszów, Poland) is a aluminosilicate mineral (the silicates). More detailed specification of the sorbents can be found in our earlier papers [66,67,68,69].

### 2.4. Microbiological Analyses

The microbiological assays of soil were made in 4 replications. The following were determined in soil samples: counts of organotrophic bacteria (Org), actinomycetes (Act) and fungi (Fun). Amounts of 10 g of soil were weighed and placed in flasks with sterile saline (90 cm^3^ 0.85% NaCl) and shaken on a laboratory shaker type 358A (Elpin, Mińsk Mazowiecki, Poland) for 30 min at 120 revolutions per minute. Dilutions of 10^−5^ and 10^−6^ were prepared to determine the counts of organotrophic bacteria and actinobacteria. Bacteria were isolated in Bunt and Rovira medium [56], actinomycetes—on Kuster and Williams medium (1971) with added nystatin and actidione [57]. In order to determine counts of fungi, dilutions of 10^−3^ and 10^−4^ were prepared. Fungi were isolated on Martin medium [58]. In order to verify the correctness of the sterilisation of media and 0.85% of NaCl during microbiological analyses, verification tests were carried out. All Petri plates with cultures were placed in an incubator (PSelecta Incudigit, Barcelona, Spain) at a temperature of 28 °C. The number of colony-forming units (cfu) was determined with a colony counter. Sums of colonies of microorganisms were calculated from the formula ISO 7218:2007/AMD 1:2013 [70]. In order to compute the colony development (CD) and ecophysiological diversity (EP) indices, colony-forming units of microorganisms were counted every day for 10 days. The CD (Formula (1)) and EP (Formula (2)) indices were determined according to De Leij et al. [71]:

CD = [N_1_/1 + N_2_/2 + N_3_/3… N_10_/10] × 100 (1)

where: N_1_, N_2_, N_3_, …, N_10_ is the sum of the ratios of the number of microbial colonies identified on individual days (1, 2, 3, …, 10) to the total number of colonies identified over the entire study period.

EP = −Σ(pi × log_10_ pi) (2)

where: pi is the ratio of the number of colonies of microorganisms identified on individual days to the total number of colonies identified over the entire study period.

### 2.5. Enzymatic Analyses

The biochemical analyses of soil included determination of the activity of dehydrogenases (Deh), catalase (Cat), acid phosphatase (Pac), alkaline phosphatase (Pal), *β*-glucosidase (Glu) and arylsulphatase (Aryl). All determinations were repeated 3 times. The analysis of the activity of dehydrogenases (EC 1.1) was carried out in line with Lenhard’s method modified by Öhlinger [59]. This method consists of the incubation of soil with water-soluble 2,3,5-triphenylthethrazole chloride (TTC), which is reduced to water-insoluble triphenylformazan (TPF). After 24 h soil incubation with calcium carbonate (in order to maintain conditions similar to natural ones), formazan is extracted from soil with ethyl alcohol. The results obtained in our study are expressed in µmol TPF kg^−1^ d.m. of soil h^−1^. The method applied to determine the activity of urease (EC 3.5.1.5) involves the incubation of soil for 24 h with 10% urea solution as a substrate. The number of ammonium ions produced was measured using the Nessler reagent. The results were expressed in mmol N-NH_4_ kg^−1^ d.m. of soil h^−1^. Determinations of the activity of urease, acid phosphatase (EC 3.1.3.2), alkaline phosphatase (EC 3.1.3.1), arylosulphatase (EC 3.1.6.1) and *β*-glucosidase (EC 3.2.1.21) were made according to the procedure developed by Alef et al. [60]. The determination of the activity of acid phosphatase and alkaline phosphatase consists of the incubation of soil in the presence of 4-nitrophenyl phosphate disodium salt 6-hydrate (PNPNa) as a substrate, which undergoes hydrolysis to 4-nitrophenol (PNP). The determination of the activity of arylsulphatase consists of the incubation of soil in the presence of potassium 4-nitrophenyl sulfate (PNS), *β*-glucosidase 4-nitrophenyl-*β*-D-glucopiranoside (PNG) as substrates. These substrates, same as in the case of phosphatases, hydrolyse to 4-nitrophenol (PNP). The activity of catalase (EC 1.11.1.6) was determined with the manganometric method according to Johnson and Temple [61]. This method consists of the decomposition of a solution of hydrogen peroxide into hydrogen and oxygen. The soil oxidation state was determined based on the reaction of decomposition of hydrogen peroxide using 0.02 M of a solution of potassium permanganate (KMnO_4_). The results obtained from these analyses were presented in mol O_2_ kg^−1^ d.m. h^−1^. Determinations of the activity of all enzymes, except catalase, were made on a Perkin–Elmer Lambda 25 spectrophotometer (Waltham, MA, USA).

### 2.6. Physicochemical and Chemical Soil Analyses

Prior to the plant growing trials, the basic characteristics of the soil had been determined, and the results of these determinations are comprised in Table 1. The textural composition of the soil was determined with a laser molecular size meter Malvern Mastersizer 2000 Laser Diffraction (Malvern, Worcestershire, UK), while the hydrolytic acidity (HAC) and exchangeable base cations (EBC) of soil were measured according to the Kappen method [55]. Based on the HAC and EBC values, the cation exchange capacity (CEC) and base saturation (BS) were calculated. The content of total N (N_tot_) was determined on a distillation unit Buchi B-324 (Buchi, Flawil, Switzerland), organic carbon (C_org_)—on a spectrophotometer Genesis 6 (Thermo Electron Corporation, Waltham, MA, USA), available phosphorus—on a spectrophotometer SQ118, potassium—Jenway PFP 7 flame photometer (Jenway LTD, Staffordshire, UK), and magnesium—on an atomic absorption spectrophotometer GBC 932AA, (GBC Scientific Equipment Australia. Exchangeable cations: K^+^, Ca^2+^ and Na^+^, were determined using a flame photometer Jenway PFP 7 (Jenway LTD, Staffordshire, UK), and Mg^2+^—on an atomic absorption spectrophotometer Agilent 280 FS AA, (Agilent Technologies, Mulgrave, Australia). pH was measured with a HI 2221 pH meter (Hanna Instruments, Washington, UK). The content of cobalt in soil was determined on an ICI (International Electrotechnical Commission) spectrometer (Thermo Scientific; Waltham, MA, USA) according to standard ISO 11047:1998 [53].

### 2.7. Statistical Analyses

The results were processed statistically in a Statistica 13.0 software package [72]. Homogenous groups were distinguished with Tukey’s test at *p* = 0.05. The analysis of variance ANOVA was run to calculate the coefficient η^2^, which reflected the contribution of particular independent variables to the shaping of dependent variables. By using the determined activity of soil enzymes, an index was calculated to display the effect of the tested sorbents and heavy metals on the activity of soil enzymes and crop yields. The following formula was employed:

IF_Ad_ or IF_Hm_ = Po/Co − 1 (3)

where:

IF_Ad_—index of the influence of the adsorbent.
IF_Hm_—index of the influence of heavy metals.
Po—the activity of enzymes in the soil or the yield of plants after the use of sorbents/contaminated with heavy metals.
Co—enzyme activity or plant yield in unpolluted soil (no heavy metals) and without sorbents.

The calculated values of the indices IF_Ad_ and IF_Hm_ were presented with the help of software RStudio v1.2.5033 (Boston, MA, USA) [73], R v3.6.2 system (Vienna, Austria) [74] and the gplots library [75] on heat maps. The lowest values of the indices were shown in yellow colour, while the highest ones were in black. The blue line depicted in the colour key and histogram shows how many times each of the data appears in the matrix used in the heat map.

## 3. Results

### 3.1. Counts of Microorganisms

**Figure 1:**
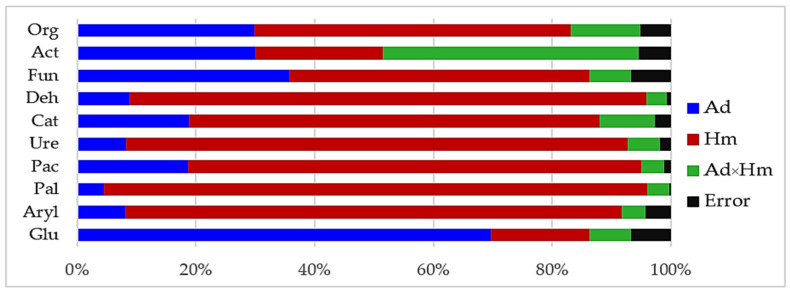
Contribution of independent variables on the number of microorganisms and activity of soil enzymes (η^2^): Ad—adsorbent, Hm—heavy metal ions. Org—organotrophic bacteria, Act—actinomycetes, Fun—fungi, Deh—dehydrogenases, Cat—catalase, Pac—acid phosphatase, Pal—alkaline phosphatase, Glu—*β*-glucosidase, Aryl—arylsulphatase.

The percent contribution of independent variables (η^2^) determined in the study exposed the significant effect of heavy metals on the counts of fungi (50.62%) and organotrophic bacteria (53.26%) (Figure 1). The application of reactive minerals and biochar induced slightly smaller changes in the counts of microorganisms. The extent of the moderating influence of the adsorbents oscillated between 29.88% for organotrophic bacteria to 35.66% for fungi.

Among the six analysed sorbents, few proved to be effective in stimulating the activity of the groups of microorganisms (Tables S1–S3). In non-polluted soil, the molecular sieve and biochar had the best effect on the count of organotrophic bacteria (Table S1) and expanded clay was most beneficial for actinobacteria (Table S2), while fungi responded positively to both expanded clay and biochar (Table S3). The stimulating effect of the reactive minerals and biochar on organotrophic bacteria was 36% and 25%, respectively, compared to 39% in the case of actinobacteria and 53% and 57% with respect to fungi. In the pots contaminated with Cd^2+^ and Co^2+^, the positive effects of the sorbents were less spectacular, as demonstrated by the identified homogenous groups. Organotrophic bacteria were the most sensitive, while actinomycetes were the most tolerant to the heavy metals applied to the soil. In the latter group of microorganisms, the molecular sieve and biochar achieved the expected role by mollifying the inhibitory impact of Cd^2+^. Both materials induced an increase in the counts of actinomycetes by 84% and 60%, respectively, compared to the control pots polluted with this heavy metal. In turn, under the pressure of Co^2+^, expanded clay showed a particular remedial ability, contributing to an increase in the count of fungi by as much as 77%.

**Figure 2:**
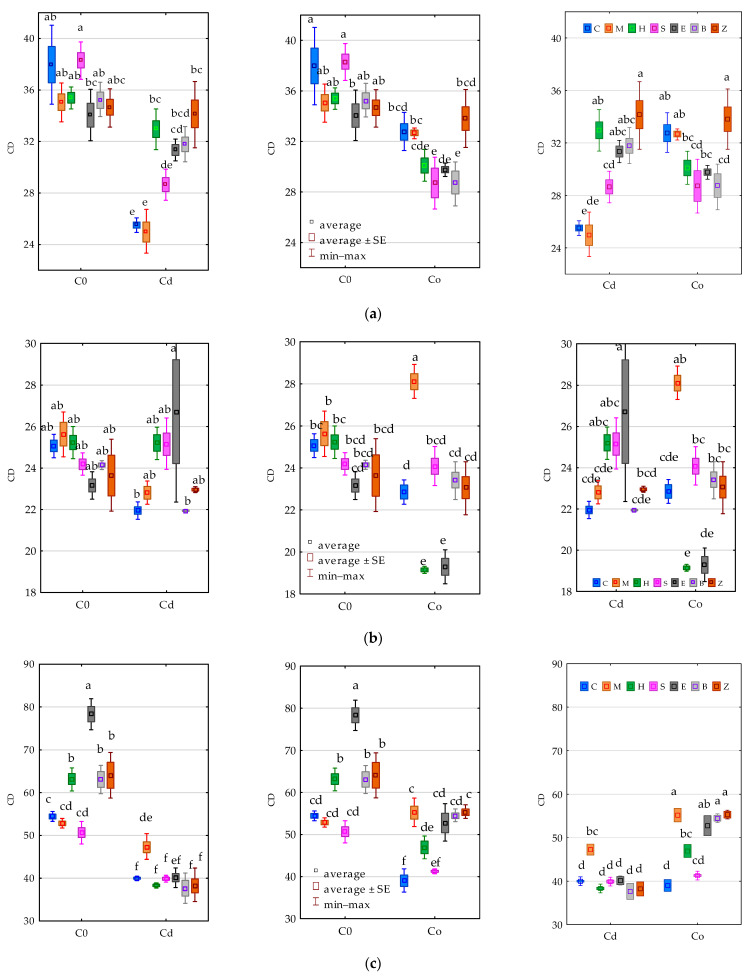
Effect of soil contamination with cadmium (Cd^2+^) and cobalt (Co^2+^) on the colony development index (CD) of (**a**) organotrophic bacteria (Org), (**b**) actinomycetes (Act) and (**c**) fungi (Fun). C0—uncontaminated soil, Cd—cadmium ion, Co—cobalt ion. C—control, M—molecular sieve, H—halloysite, S—sepiolite, E—expanded clay, B—biochar, Z—zeolite. Homogeneous groups denoted with letters (a–f) were calculated separately for each pair of independent variables.

Values of the colony development (CD) indices obtained for the analysed groups of microorganisms revealed the inhibitory effect of both Cd^2+^ and Co^2+^ on this parameter (Figure 2). Although sepiolite stimulated the multiplication of organotrophic bacteria, it was ineffective in alleviating the negative influence of the heavy metals applied to the soil, in which it resembled the other tested sorbents.

A reverse dependence was noted in the case of actinomycetes. None of the sorbents stimulated the multiplication of this group of microorganisms in soil free from heavy metals. On the other hand, the complication of the exposure to Cd^2+^ and soil remediation with expanded clay or soil contamination with Co^2+^ and the application of a molecular sieve contributed to a rise in the CD value for actinomycetes. In the pots unpolluted with heavy metals, expanded clay also induced the multiplication of fungi (CD = 78.329). None of the sorbents managed to alleviate the negative effects of such spectacular pressure by Cd^2+^ and Co^2+^. It is worth underlining that the broader pool of applied reactive minerals, including a molecular sieve, expanded clay and zeolite as well as biochar was more effective in constraining the inhibitory impact of Co^2+^ than that of Cd^2+^ on the multiplication rate of fungi.

**Figure 3:**
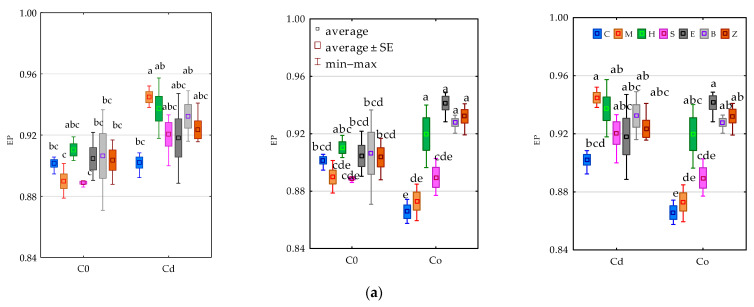

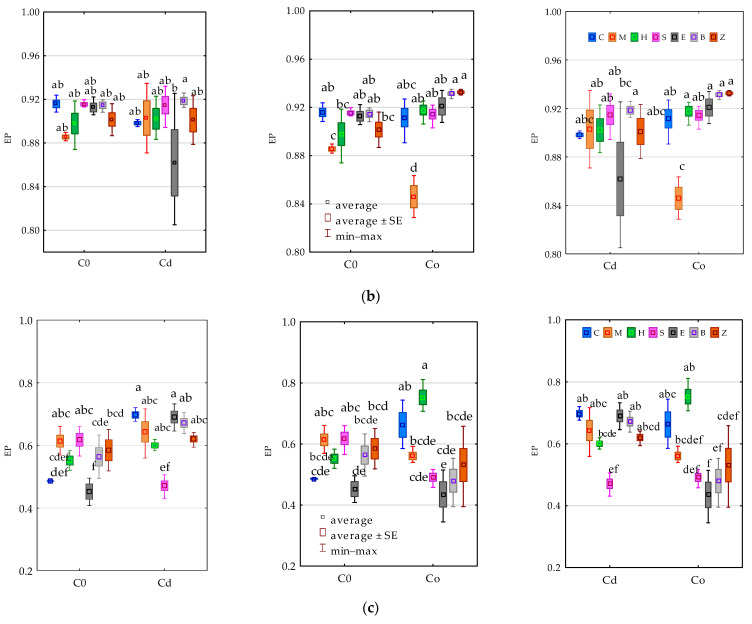
Effect of soil contamination with cadmium (Cd^2+^) and cobalt (Co^2+^) on the colony diversity index (EP) of (**a**) organotrophic bacteria (Org), (**b**) actinomycetes (Act) and (**c**) fungi (Fun). C0—uncontaminated soil, Cd—cadmium ion, Co—cobalt ion. C0—uncontaminated soil, Cd—cadmium ion, Co—cobalt ion. C—control, M—molecular sieve, H—halloysite, S—sepiolite, E—expanded clay, B—biochar, Z—zeolite. Homogeneous groups denoted with letters (a–f) were calculated separately for each pair of independent variables.

The response of microorganisms to the tested factors was also traced through the prism of the ecophysiological diversity (EP) values (Figure 3). In the pots free from contamination with the heavy metals, none of the tested sorbents had a positive effect on the EP of organotrophic bacteria comparable to that obtained with the molecular sieve in soil exposed to 100 mg Cd^2+^ kg^−1^ d.m. of soil (EP = 0.945). In turn, the pressure of 100 mg Co^2+^ kg^−1^ d.m. of soil on this parameter was mollified by halloysite, expanded clay, zeolite and biochar. It is worth emphasising that the response of actinomycetes to the compilation of soil contamination with Co^2+^ and its remediation with a molecular sieve was completely opposite. The ecophysiological diversity index in this pool of objects was observed to have decreased. Among fungi, which were characterised by the lowest EP values, a comparable trend was observed in the objects with sepiolite in the soil polluted with Cd^2+^ and Co^2+^.

### 3.2. Enzyme Activity

**Figure 4:**
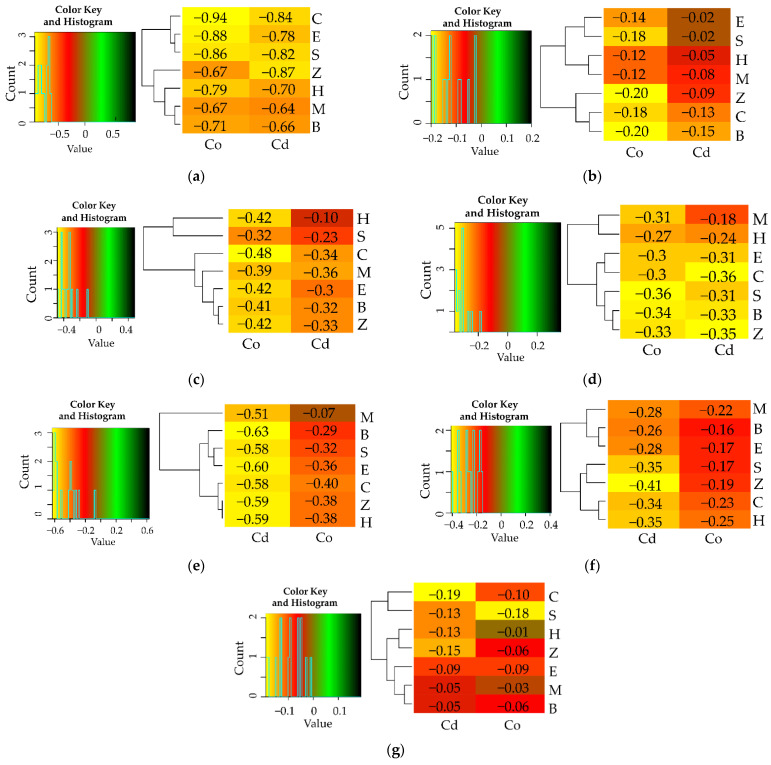
Indexes of the influence of heavy metals on the activity of enzymes (IF_Hm_). (**a**) dehydrogenases (Deh), (**b**) catalase (Cat), (**c**) urease (Ure), (**d**) acid phosphatase (Pac), (**e**) alkaline phosphatase (Pal), (**f**) arylsulfatase (Aryl), (**g**) *β*-glucosidase (Glu). M—molecular sieve, H—halloysite, S—sepiolite, E—expanded clay, B—biochar, Z—zeolite, C—soil not contamination, Cd—ion Cd^2+^, Co—Co^2+^.

The differentiated response of soil enzymes, demonstrated by the $\eta^{2}$ values was another manifestation of the extent of biotic stress induced by soil contamination with Cd^2+^ and Co^2+^ (Figure 1). Having assessed the percentage contribution of the variables, the response of enzymes to the applied adsorbents was evaluated and arranged in the following order: Glu > Cat > Pac > Deh > Aryl = Ure > Pal. The heavy metals had the highest moderating effect on Pal (91%), Deh (86%) and Ure (84%), and the weakest one—on the activity of Glu (17%). Co^2+^ proved to be less toxic to Deh and Ure (Tables S4 and S6), and Cd^2+^ was less toxic to Pal (Table S7). The activity of enzymes declined by 94% (Deh) and 48% (Ure) under the pressure of Co^2+^ and by 58% (Pal) under the influence of Cd^2+^ relative to the control objects. With respect to the remaining enzymes, the inhibitory effect of both heavy metals was similar (Tables S5 and S8–S10). The index of the effect of heavy metals on the activity of soil enzymes (IF_Hm_) attested to the particular sensitivity of Deh, Pal and Ure to being exposed to Cd^2+^ and Co^2+^ (Figure 4). Values of the IF_Hm_ also distinguished the most stable enzymes in soil exposed to the pressure of the heavy metals, which were *β*-glucosidase and catalase.

**Figure 5:**
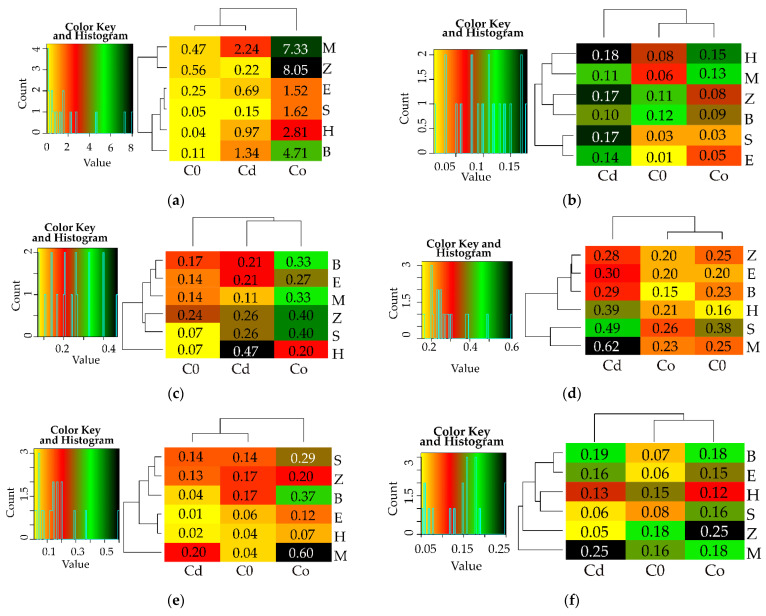

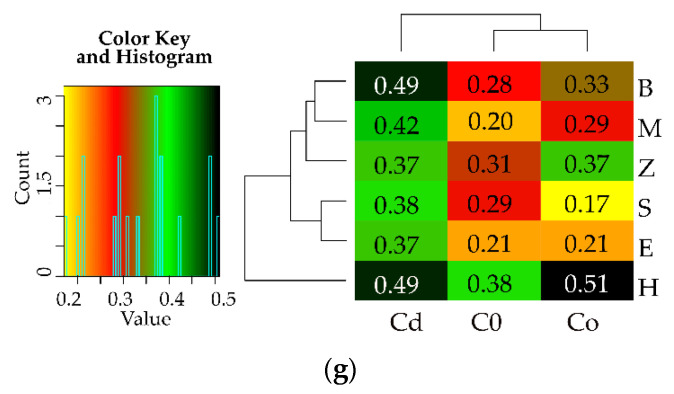
Indexes of the influence of sorbents on the activity of enzymes (IF_Ad_). (**a**) dehydrogenases (Deh), (**b**) catalase (Cat), (**c**) urease (Ure), (**d**) acid phosphatase (Pac), (**e**) alkaline phosphatase (Pal), (**f**) arylsulfatase (Aryl), (**g**) *β*-glucosidase (Glu), M—molecular sieve, H—halloysite, S—sepiolite, E—expanded clay, B—biochar, Z—zeolite, C—soil not contamination, Cd—ion Cd^2+^, Co—Co^2+^.3.3. The Reaction Sunflower (Helianthus annuus) to Cd^2+^ and Co^2+^.

Based on the values of the index showing the effect of sorbents on the activity of enzymes (IF_Ad_), the sorbents which improved the condition of the soil were identified (Figure 5). However, none of the adsorbents applied restored the equilibrium of the soil after it had been polluted with heavy metals. Zeolite in unpolluted soil stimulated the activity of Deh, Ure, Aryl and Pal, inducing its increase by 56%, 24%, 18% and 17%, respectively, in comparison to the control objects. Sepiolite and halloysite raised the activity of Pac and Glu by 38%, whereas biochar contributed to a rise in the activity of catalase by 12%. When reviewing the remediation potential of the tested adsorbents, it was concluded that all the reactive minerals and biochar only alleviated the inhibitory impact of the heavy metals, except expanded clay added to the soil exposed to the pressure of Cd^2+^, but did not completely eliminate the impact of heavy metals. The extent of their influence varied. Regardless of the metal applied to soil, the molecular sieve and biochar decreased the toxic effect of Cd^2+^ and Co^2+^ on the activity of dehydrogenases. However, the highest value of IF_Ad_ = 8.048 was obtained in the soil polluted with Co^2+^ and amended with zeolite. Halloysite caused an increase in the activity of urease. The activity of this enzyme in the objects with Co^2+^ contaminated soil was also stimulated by zeolite and sepiolite. Higher effectiveness of these three sorbents was also noted regarding catalase, but only in the soil contaminated with Cd^2+^. In this pool of objects, the molecular sieve proved to be the remediation factor that mostly enhanced the activity of Pac and Pal. The adsorbents which were ineffective in stimulating the activity of particular soil enzymes were also identified. The objects polluted with Co^2+^ halloysite did not significantly stimulate the activity of Ure, Pal and Aryl, sepiolite—Cat and Glu, expanded clay—Deh, and biochar—Pac. In the soil contaminated with Cd^2+^, the expected effects were not achieved by expanded clay as regards Pal and Glu, and zeolite as regards Pac and Arul. Sepiolite, biochar and molecular sieve generated the lowest values of the IF_Ad_ for Deh (IF_Ad_ = 0.15), Cat (IF_Ad_ = 0.10) and Ure (IF_Ad_ = 0.11), respectively.

**Figure 6:**
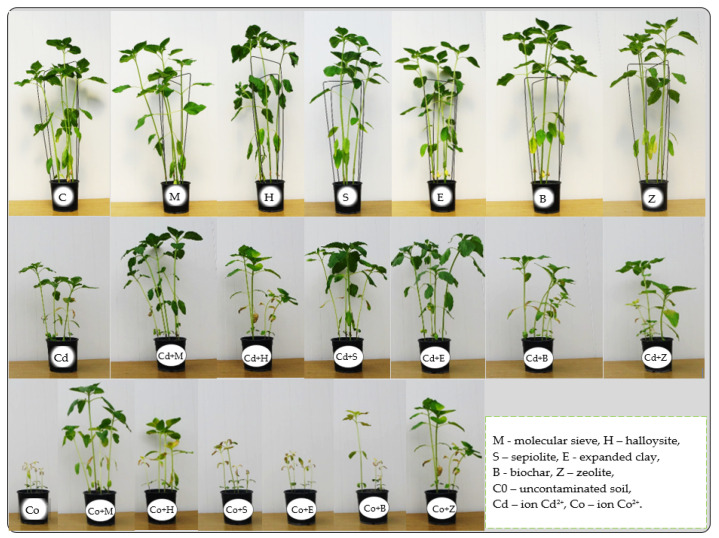
*Helianthus annuus* L. in the BBCH phase 34.

**Figure 7:**
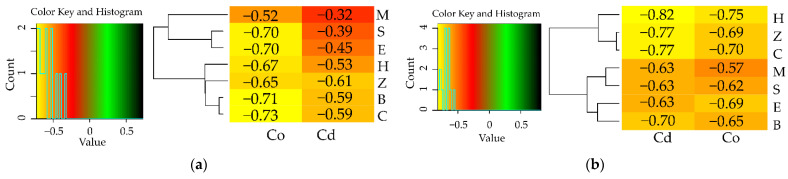
Index of the influence of heavy metals (IF_Hm_) on the yield of (**a**) aboveground parts, (**b**) roots of plants.

**Figure 8:**
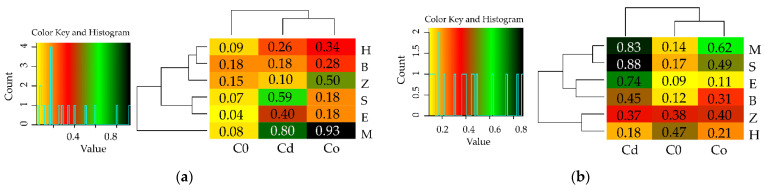
Index of the influence of adsorbents (IF_Ad_) on the yield of the part (**a**) aboveground plants, (**b**) roots of plants.

A more comprehensive character of this study was achieved when the response of the sunflower to the pressure of Cd^2+^ and Co^2+^ was analysed, and the usefulness of the tested adsorbents in remediation of soils polluted with heavy metals was verified, including the yields of the test plant (Tables S11 and S12, Figure 6). Co^2+^ had a stronger inhibitory effect on the growth and development of the sunflower’s aboveground parts, whose yields were lower by as much as 73% under the pressure of this metal relative to the control, in comparison to a 59% lower yield in soil polluted with Cd^2+^ (Table S11). Out of the six adsorbents applied to the soil not exposed to heavy metals, biochar and zeolite induced an increase in the crop’s aboveground parts by 18% and 15%, respectively, relative to the control. Notwithstanding this, both adsorbents mitigated the least the toxic effect of Cd^2+^ on this parameter. The values of the index illustrating the impact of the heavy metals on the sunflower yields (IF_Hm_) revealed that the extent of the negative influence of both Cd^2+^ (IF_Hm_ = −0.32) and Co^2+^ (IF_Hm_ = −0.52) was the smallest in the compilation with the molecular sieve (Figure 7a). Determination of the usefulness of adsorbents based on the IF_Ad_ exposed their potential to alleviate the toxic effect of the heavy metals on the yield of the cultivated crop (Figure 8a). However, this potential was varied and dependent on the type of heavy metal. The lowest value of the IF_Ad_ in soil exposed to the pressure of Cd^2+^ was noted for zeolite (IF_Ad_ = 0.10) and to Co^2+^—for sepiolite and expanded clay (IF_Ad_ = 0.18).

When evaluating the response of sunflowers through the prism of the mass of sunflower roots, different relationships emerged (Table S12). Cd^2+^ proved to be a stronger inhibitor to the crop’s root system. The application of this heavy metal generated the root mass lower by 77%, compared to 70% in response to Co^2+^, relative to the unpolluted objects. The usefulness of the adsorbents for remediation of soil was traced following the response of the sunflower roots, which displayed two significant relationships: halloysite induced an increase in the root biomass by 44% and zeolite by 38% in unpolluted soil (1) and according to the IF_Hm_ values, none of these adsorbents was as effective in the alleviation of the negative impact of the heavy metals (2) (Figure 6). The pool of the objects contaminated with Cd^2+^, sepiolite, expanded clay, and molecular sieve generated the lowest values of the IF_Hm_ = −0.63, and the molecular sieve was the most effective in alleviating the toxic effect of cobalt in Co^2+^ contaminated soil (IF_Hm_ = −0.571). Having traced the values of the index showing the effect of sorbents (IF_Ad_) on the development of the sunflower’s root system, we were able to identify the reactive minerals that played the least significant role in the simulation of the root’s growth (Figure 7b). These include expanded clay in unpolluted soil (IF_Ad_ = 0.09) and in soil exposed to Co^2+^ (IF_Ad_ = 0.11), as well as halloysite in the objects contaminated with Cd^2+^ (IF_Ad_ = 0.18). Co^2+^ disturbed the synthesis of chlorophyll spectacularly, as evidenced by the low values of the greenness index (4.45) (Table 2). However, this process was significantly mollified by the molecular sieve, same as in the objects polluted with Cd^2+^, although the inhibitory effect of this metal (SPAD = 21.62) was not as large as the one produced by Co^2+^.

## 4. Discussion

### 4.1. Microorganisms

Contamination of soil with Co^2+^ or with Cd^2+^ caused undesirable changes in the soil’s microbiological parameters. They included depressed counts of organotrophic bacteria and, to a lesser extent, of fungi and actinomycetes. Admittedly, negative effects of the impact of these heavy metals on the soil microbiome were expected, as suggested by reports on the damage of the integrity of the cell membrane in microorganisms, inactivation or oxidation of cellular enzymes, protein denaturation, or inhibition of the process of transcription by heavy metals [76]. However, it is known that one of the common adaptation strategies of microorganisms relies on the extracellular production of metallophores and polysaccharides [77] and on horizontal gene transfer, where mobile genetic elements, such as transposons and integrons, play an important role [78]. Co^2+^ proved to be a stronger inhibitor of the multiplication of actinomycetes and fungi than Cd^2+^. Considering the fact that Co^2+^ is absorbed by microorganisms in order to produce enzyme cofactors (cobamide) participating in the metabolic reactions essential for live cells [79], it shows affinity to thiol groups of peroxiredoxins aggregated after hyperoxidation to protect proteins from denaturation [80], opposite tendencies were expected. Kosiorek and Wyszkowski [81] also report that Co^2+^ may retard or stimulate the division of cells depending on its dose. However, the correlations obtained in this study correspond well to the research results achieved by Wyszkowska et al. [44], Boros-Lajszner et al. [82] and Zaborowska et al. [83]. It has been demonstrated that the toxicity of Co^2+^ in the periplasm regulated by the cytochromes GSU_1538_ and GSU_2513_ is associated with the diffusion of this metal through non-selective pores of the external membrane, leading to the rapid accumulation of Co^2+^ in the periplasm space, thereby interfering with basic cellular functions [84]. The toxicity of Cd^2+^ towards microorganisms raises fewer questions as it has been confirmed by numerous scientific reports [44,82,85,86]. Furthermore, Gunina and Kuzyakow [87] observed that Cd^2+^ pressure caused a rapid rate of the uptake of sugars from the soil by microorganisms, which stimulated microbial activity and accelerated the decomposition of SOM. The depressed availability of organic matter could evoke a strong stress response in microorganisms. Thus, the application of adsorbents as a remediation factor is highly justified, particularly the use of a molecular sieve and biochar, which not only induced the growth of organotrophic bacteria but also mollified the toxic effect of Cd^2+^. In addition, the molecular sieve enhanced the rate of multiplication of actinomycetes and had a beneficial influence on the ecophysiological diversity of organotrophic bacteria. The effectiveness of biochar was most probably connected with its unique parameters responsible for the adsorption potential of heavy metals in arable soils, such as surface loads and pore spaces correlated with the content of nutrients and the ability to exchange cations (CEC) [88,89]. In their studies, Strachel et al. [66] and Steiner et al. [90] also demonstrated that biochar enhanced the activity of the soil microbiome. A molecular sieve owes its effectiveness to silanol groups (Si-OH) localised on the surface of the mineral, which can strongly immobilise Cd^2+^ in soil [91]. Strachel et al. [66] showed that a molecular sieve, same as biochar, had the strongest positive effect on the counts of organotrophic bacteria and actinomycetes, which supports the results of our experiment. The effect produced by expanded clay should not be neglected either, as it alleviated the negative impact of Co^2+^ on counts of all analysed groups of microorganisms and contributed to a more intensive proliferation of both fungi and actinomycetes. The usefulness of expanded clay as a bioremediation substance arises from the double porosity of this mineral, both the porous core and the intercrystallite space [92]. Expanded clay is increasingly often used as a material for amending wetlands owing to its ability to remove phosphates and its structure that supports the activity of the microbiome [93].

### 4.2. Soil Enzymes

Being able to conduct a reliable analysis of the potential of microorganisms in soil transformations requires adequate quantification of the activity of soil enzymes [94]. Priority is given to soil ecosystems with distorted homeostasis. The response of the analysed soil enzymes exposed the negative effect of Co^2+^ and Cd^2+^ on the condition of the soil. Among the seven analysed soil enzymes, dehydrogenases, urease, acid, and alkaline phosphatase appeared to be particularly sensitive to the pressure of both heavy metals. Different responses of the broad pool of enzymes to the two heavy metals reflected the complexity of the forms in which these enzymes occur in soil. Extracellular enzymes are released from cells of microorganisms undergoing lysis. They are also associated with enzyme-substrate complexes or are complex with organic matter through the process of adsorption or copolymerisation. Some enzymes also bind to condensed tannins or are adsorbed on clay materials [95]. The highest sensitivity of dehydrogenases to the toxicity of Co^2+^ and Cd^2+^ could be attributed to the fact that these enzymes are the basic elements of the enzymatic system of all living microorganisms [96]. It is also known that one of the key abiotic environmental factors moderating the activity of all enzymes is the soil pH [97]. A rise in this parameter can contribute to the damage of ionic and hydrogen bonds in the active centre of dehydrogenases [98]. In turn, the negative effect of Co^2+^ on the activity of urease could be a consequence of interactions of complexes containing this metal with sulfhydryl groups of cysteines, histidine nitrogen atoms or oxygen atoms of glutamic acid residues of urease amino acids [99].

While the application of adsorbents did not restore the equilibrium of soil, some of these substances stood out by their ability to alleviate the toxic effect of heavy metals. Apart from a molecular sieve and biochar, which determined the activity of Deh, other reactive minerals were distinguished as being able to minimise the adverse impact of Co^2+^ on the activity of Deh and Cat (zeolite) or Ure (halloysite, sepiolite and zeolite), and were also effective against the negative influence of Cd^2+^ in soil. The effectiveness of a molecular sieve and zeolite might be attributed to the homogenous structure of these sorbents [100] and heavy metal binding affinity [101]. Of importance for the bioremediation potential is the three-dimensional skeleton of tetrahedral bonds [SiO_4_]^4−^ and [AlO_4_]^5−^ of zeolite studded with spaces for cation exchange, which creates the catalytic characteristics of this material and facilitates the process of adsorption, desorption and ionic exchange [102]. Boros-Lajszner et al. [69] also implicate an important role of zeolite in the remediation of soil contaminated with heavy metals. In turn, Strachel et al. [66] maintain that halloysite is a significant stimulator of Pac, Deh and Ure. Yuan et al. [103] claim that this clay mineral is effective in absorbing pollutants by creating surface complexes, which resemble sepiolite, which causes changes in the activity of alkaline phosphatase, *β*-glucosidase [66], urease [104] and dehydrogenase [105].

### 4.3. Sunflower (Helianthus annuus L.) against Soil Contamination with Co^2+^ and Cd^2+^

The choice of the sunflower (*Helianthus annuus*) as a test plant was dictated by its ability to remove heavy metals from soils [106,107]. It was demonstrated that both Co^2+^ and Cd^2+^ significantly disturbed the yielding of the test plant. Similar relationships were noted by Forte and Mutiti in soil polluted by Cd^2+^ [106]. Under the pressure of 200 mg Cd^2+^ kg^−1^ d.m. of soil, the dry matter of the stem decreased by 64.30%, and that of the root was lowered by 80.80%. Higher toxicity of Co^2+^ towards the sunflower is certainly a consequence of the heavy metal interfering with the structure and number of chloroplasts, damage to the morphology of plastids and inhibition of the synthesis of the RNA as well as amounts of the RNA and DNA through the modification of the activity of endo- and exonucleases [108]. In turn, Cd^2+^ depresses the activity of the oxidative enzymes CAT or SOD [36]. Stimulation of ROS in a plant induces an increase in the synthesis of the mitogen-activated protein (MAP) kinase [109], and the Cd^2+^ stimulated synthesis of the radicals H_2_O_2_ and OH eventually leads to the necrosis of plant tissues [110]. Due to the competition with Cd^2+^, the uptake and, consequently, concentrations of K, Ca, Mn, Zn, Cu and Fe in plants decrease, causing their deficiencies [111]. This study proves that the response of the sunflower to the contamination of soil with heavy metals, including Co^2+^, is manifested by the yellowing of leaves correlated with the decreasing content of chlorophyll, as evidenced by the SPAD values. According to Palit et al. [108], the role of this metal in the process of photosynthesis is controversial. However, the toxic effect of Co^2+^ on this parameter is manifested by the inhibition of the acceptor PS_2_, thereby slowing down the Hill reaction and by the reduced export of photo assimilators. Furthermore, Cd^2+^ has an inhibitory effect on photosynthesis by retarding the activity of the Calvin cycle enzymes. In addition, it induces changes in the lipid composition of thylakoid membranes [112].

The molecular sieve manifested its remediation properties in the soil contaminated with Co^2+^ and Cd^2+^. Interestingly, biochar and zeolite significantly induced the growth and development of the sunflower in unpolluted soil. However, the compilation of these sorbents did not yield the expected results, which would be a higher yield of this crop. This could be explained by the fact that biochar produced by pyrolysis may contain small amounts of toxic substances, usually As, Cr, Pb and Cd. Hence, before its application as a biosorbent, it is recommended to leach metals from biochar [113].

## 5. Conclusions

Co^2+^ and Cd^2+^ significantly interfered with the soil’s microbiome, distorting its balance. Organotrophic bacteria proved to be the least sensitive to soil contamination with Cd^2+^, while fungi and actinomycetes were least sensitive to the pollution of soil with Co^2+^. The inhibitory effect of Cd^2+^ was also manifested by the decreasing CD values of actinomycetes and organotrophic bacteria, thereby implicating a change in the structure of these groups of microorganisms from r strategists to K strategists. Dehydrogenases and urease were most sensitive to the pressure of Cd^2+^, while dehydrogenases and alkaline phosphatase—to Co^2+^. The response of *Helianthus annuus* to soil pollution with heavy metals consisted in the significantly disturbed growth and development of this plant, both the aboveground parts and the roots, although Co^2+^ inhibited the synthesis of chlorophyll more spectacularly. None of the tested adsorbents restored the balance of soil after the contamination with the heavy metals, although all of them, to a different extent, alleviated the toxic influence of the metals on the soil’s microbiological activity and biochemical activity and yields of the sunflower. A molecular sieve and biochar demonstrated remediation potential towards microorganisms and enzymatic activity. The activity of soil enzymes was also stimulated by zeolite and sepiolite. The use of a molecular sieve induced an increase in the biomass of *Helianthus annuus* of both aboveground organs and roots. Having analysed all the parameters included in this experiment, it was concluded that a molecular sieve might be the most useful sorbent in the remediation of soils polluted with Co^2+^ and Cd^2+^. The obtained results set the direction for further research, which should be carried out under environmental conditions. The use of sorbents in the reclamation of Cd^2+^ and Co^2+^ contaminated soils should also be preceded by an economic calculation in connection with the impact of these pollutants on the environment.

## Figures and Tables

**Table 1:** Some properties of the soil and compost used in the experiment.

Abbreviation	Properties	Unit	Soil	Methodical Literature
Chemical and physicochemical properties				
N_tot_	total nitrogen	g kg^−1^ d.m.	1.07	[49]
C_org_	organic carbon	g kg^−1^ d.m.	14.69	[50]
P	phosphorus	mg kg^−1^ d.m.	166.72	[51]
K	potassium	mg kg^−1^ d.m.	171.31	[51]
Mg	magnesium	mg kg^−1^ d.m.	443.21	[52]
Cd	cadmium	mg kg^−1^ d.m.	0.56	[53]
Co	cobalt	mg kg^−1^ d.m.	7.21	[53]
pH	pH_KCl_—soil reaction	-	6.00	[54]
EBC	sum of exchangeable base cations	mmol (+) kg^−1^ d.m.	145.00	[55]
HAC	hydrolytic acidity	mmol (+) kg^−1^ d.m.	13.50	[55]
CEC	cation exchange capacity	mmol (+) kg^−1^ d.m.	158.50	[55]
ACS	alkaline cation saturation	%	91.49	[55]
Microorganisms number per 1 kg d.m.				
Org	organotrophic bacteria	cfu	18.192 × 10^9^	[56]
Act	actinomyces	cfu	11.931 × 10^9^	[57]
Fun	fungi	cfu	5.374 × 10^7^	[58]
Enzymatic activity per 1 kg d.m. h^−1^				
Deh	dehydrogenases	µmol TPF	3.124	[59]
Cat	catalase	mol O_2_	0.304	[60]
Ure	urease	mmol N-NH_4_	0.492	[61]
Pac	acid phosphatase	mmol PN	1.168	[61]
Pal	alkaline phosphatase	mmol PN	1.014	[61]
Glu	*β*-glucosidase	mmol PN	0.492	[61]
Aryl	arylsulphatase	mmol PN	0.140	[61]

**Table 2:** Greenness index (SPAD).

Object	C0	Cd	Co
Control	35.988 ^ab^	21.625 ^d–f^	4.450 ^g^
Molecular sieve	34.713 ^a^	29.563 ^bc^	28.313 ^b–d^
Halloysite	38.638 ^a^	19.588 ^ef^	21.325 ^d–f^
Sepiolite	35.175 ^ab^	25.413 ^c–e^	22.963 ^c–e^
Expanded clay	35.238 ^ab^	24.275 ^c–e^	14.563 ^f^
Biochar	39.138 ^a^	22.975 ^c–e^	19.438 ^ef^
Zeolite	33.488 ^a^	20.213 ^ef^	14.525 ^f^

Homogeneous groups denoted with letters (^a–f^).

## Data Availability

Data are available by contacting the authors.

## Supplementary Materials

The following supporting information can be downloaded at: https://www.mdpi.com/article/10.3390/ma15165738/s1, Table S1: The number of organotrophic bacteria, 10^9^ cfu kg^−1^ d.m. of soil; Table S2: The number of actinomycetes, 10^9^ cfu kg^−1^ d.m. of soil; Table S3: The number of fungi, 10^7^ cfu kg^−1^ d.m. of soil; Table S4: Dehydrogenases activity, µmol TPF h^−1^ kg^−1^ d.m. of soil; Table S5: Catalase activity, mol O_2_ h^−1^ kg^−1^ d.m. of soil; Table S6: Urease activity, mmol N-NH_4_ h^−1^ kg^−1^ d.m. of soil; Table S7: Alkaline phosphatase activity, mmol PNP kg^−1^ h^−1^ kg^−1^ d.m. of soil; Table S8: Acid phosphatase activity, mmol PNP h^−1^ kg^−1^ d.m. of soil; Table S9: Arylsulfatase activity, mmol PNS h^−1^ kg^−1^ d.m. of soil; Table S10: *β*-glucosidase activity, mmol PNG h^−1^ kg^−1^ d.m. of soil; Table S11: Yield of aboveground parts of sunflower (*Helianthus annuus*), g d.m. on pot^−1^; Table S12: Sunflower (*Helianthus annuus*) root yield, g d.m. pot^−1^.

## Abbreviations

M—molecular sieve, H—halloysite, S—sepiolite, E—expanded clay, B—biochar, Z—zeolite. Cd—ion Cd^2+^, Co—ion Co^2+^, SPAD—the leaf greenness index; Org—organotrophic bacteria; Act—actinomycetes; Fun—fungi; Deh—dehydrogenases; Cat—catalase; Ure—urease; Pac—acid phosphatase; Pal—alkaline phosphatase; Aryl—arylsulfatase, Glu—*β*-glucosidase; CD—colony development index; EP—ecophysiological diversity index.

## References

[1] Barra Caracciolo A., Terenzi V. (2021). Rhizosphere microbial communities and heavy metal. Microorganisms.

[2] Karlen D.L., Rice C.W. (2015). Soil degradation: Will humankind ever learn?. Sustainability.

[3] Hossain A., Krupnik T.J., Timsina J., Mahboob M.G., Chaki A.K., Farooq M., Fahad S., Hasanuzzaman M., Alam M., Ullah H., Saeed M., Ali Khan I., Adnan M. (2020). Agricultural land degradation: Processes and problems undermining future food security. Environment, Climate, Plant and Vegetation Growth.

[4] Kopittke P.M., Menzies N.W., Wang P., McKenna B.A., Lombi E. (2019). Soil and the intensification of agriculture for global food security. Environ. Int..

[5] Lichtfouse E., Navarrete M., Debaeke P., Souchere V., Alberola C., Menassieu J. (2009). Agronomy for sustainable agriculture. A review. Agron. Sustain. Dev..

[6] Genchi G., Sinicropi M.S., Lauria G., Carocci A., Catalano A. (2020). The effects of cadmium toxicity. Int. J. Environ. Res. Public Health.

[7] Awan S.A., Ilyas N., Khan I., Raza M.A., Rehman A.U., Rizwan M., Rastogi A., Tariq R., Brestic M. (2020). *Bacillus siamensis* reduces cadmium accumulation and improves growth and antioxidant defense system in two wheat (*Triticum aestivum* L.) varieties. Plants (Basel, Switz.). Plants.

[8] Ali H., Khan E., Ilahi I. (2019). Environmental chemistry and ecotoxicology of hazardous heavy metals: Environmental persistence, toxicity, and bioaccumulation. J. Chem..

[9] Xu J., Hu C., Wang M., Zhao Z., Zhao X., Cao L., Lu Y., Cai X. (2022). Changeable effects of coexisting heavy metals on transfer of cadmium from soils to wheat grains. J. Hazard. Mater..

[10] Kusvuran S., Kiran S., Ellialtioglu S.S. (2016). Antioxidant enzyme activities and abiotic stress tolerance relationship in vegetable crops. Abiotic and Biotic Stress in Plants—Recent Advances and Future Perspectives.

[11] Mashhadikhan S., Amooghin A.E., Sanaeepur H., Shirazi M.M.A. (2022). A critical review on cadmium recovery from wastewater towards environmental sustainability. Desalination.

[12] Rigby H., Smith S.R. (2020). The significance of cadmium entering the human food chain via livestock ingestion from the agricultural use of biosolids, with special reference to the UK. Environ. Int..

[13] European Commission (2020). Study on the Review of the List of Critical Raw Materials-Final Report.

[14] Casado M., Anawar H.M., Garcia-Sanchez A., Santa Regina I. (2008). Cadmium and zinc in polluted mining soils and uptake by plants (El Losar mine, Spain). Int. Environ. Pollut..

[15] Thompson J., Bannigan J. (2008). Cadmium: Toxic effects on the reproductive system and the embryo. Reproduct. Toxicol..

[16] Hayat M.T., Nauman M., Nazir N., Ali S., Bangash N. (2019). Environmental hazards of cadmium: Past, present, and future. Cadmium Toxicity and Tolerance in Plants.

[17] Genchi G., Carocci A., Lauria G., Sinicropi M.S., Catalano A. (2020). Nickel: Human health and environmental toxicology. Int. J. Environ. Res. Public Health.

[18] ATSDR (2017). Substance Priority List (SPL).

[19] Dehaine Q., Tijsseling L.T., Glass H.J., Törmänen T., Butcher A.R. (2021). Geometallurgy of cobalt ores: A review. Miner. Eng..

[20] Johnson D.B., Dybowska A., Schofield P.F., Herrington R.J., Smith S.L., Santos A.L. (2020). Bioleaching of arsenic-rich cobalt mineral resources, and evidence for concurrent biomineralisation of scorodite during oxidative bio-processing of skutterudite. Hydrometallurgy.

[21] USGS (2020). Mineral Commodity Summaries—Cobalt. Reston, Virginia.

[22] Darton Commodities Ltd. (2020). Cobalt Market Review 2019–2020. Guildford. https://www.dartoncommodities.co.uk/cobalt/.

[23] Gil A., Santamaría L., Korili S.A., Vicente M.A., Barbosa L.V., de Souza S.D., Marçal L., de Faria E.H., Ciuffi K.J. (2021). A review of organic-inorganic hybrid clay based adsorbents for contaminants removal: Synthesis, perspectives and applications. J. Environ. Chem. Eng..

[24] Oustriere N., Marchand L., Lottier N., Motelica M., Mench M. (2017). Long-term Cu stabilization and biomass yields of Giant reed and poplar after adding a biochar, alone or with iron grit, into a contaminated soil from a wood preservation site. Sci. Total Environ..

[25] Park J.H., Lamb D., Paneerselvam P., Choppala G., Bolan N., Chung J.W. (2011). Role of organic amendments on enhanced bioremediation of heavy metal (loid) contaminated soils. J. Hazard. Mater..

[26] Cao F., Lian C., Yu J., Yang H., Lin S. (2019). Study on the adsorption performance and competitive mechanism for heavy metal contaminants removal using novel multipore activated carbons derived from recyclable long-root *Eichhornia crassipes*. Bioresour. Technol..

[27] Belviso C., Cavalcante F., Ragone P., Fiore S. (2012). Immobilization of Zn and Pb in polluted soil by *in-situ* crystallization zeolites from fly ash. Water Air Soil Pollut..

[28] Okoye E.A., Bocca B., Ruggieri F., Ezejiofor A.N., Nwaogazie I.L., Domingo J.L., Rovira J., Frazzoli C., Orisakwe O.E. (2021). Metal pollution of soil, plants, feed and food in the Niger Delta, Nigeria: Health risk assessment through meat and fish consumption. Environ. Res..

[29] Fu Z.S., Xi S.H. (2020). The effects of heavy metals on human metabolism. Toxicol. Mech. Methods.

[30] Huang H., Wang J., Zhang J., Cai J., Pi J., Xu J.F. (2021). Inspirations of cobalt oxide nanoparticle based anticancer therapeutics. Pharmaceutics.

[31] Manoj S.R., Karthik C., Kadirvelu K., Arulselvi P.I., Shanmugasundaram T., Bruno B., Rajkumar M. (2020). Understanding the molecular mechanisms for the enhanced phytoremediation of heavy metals through plant growth promoting rhizobacteria: A review. J. Environ. Manag..

[32] Amari T., Ghnaya T., Abdelly C. (2017). Nickel, cadmium and lead phytotoxicity and potential of halophytic plants in heavy metal extraction. S. Afr. J. Bot..

[33] Igiri B.E., Okoduwa S.I.R., Idoko G.O., Akabuogo E.P., Adeyi A.O., Ejiogu I.K. (2018). Toxicity and bioremediation of heavy metals contaminated ecosystem from tannery wastewater: A review. J. Toxicol..

[34] Levy D.B., Barbarick K.A., Siemer E.G., Sommers L.E. (1992). Distribution and partitioning of trace metals in contaminated soils near Leadville, Colorado. J. Environ. Qual..

[35] Hussain A., Rizwan M., Ali S., Rehman M.Z., Qayyum M.F., Nawaz R., Ahmad A., Asrar M., Ahmad S.R., Alsahli A.A. (2021). Combined use of different nanoparticles effectively decreased cadmium (Cd) concentration in grains of wheat grown in a field contaminated with Cd. Ecotoxicol. Environ. Saf..

[36] Qianqian M., Haider F.U., Farooq M., Adeel M., Shakoor N., Jun W., Jiaying X., Wang X.W., Panjun L., Liqun C. (2022). Selenium treated Foliage and biochar treated soil for improved lettuce (*Lactuca sativa* L.) growth in Cd-polluted soil. J. Clean. Prod..

[37] Ojuederie O.B., Babalola O.O. (2017). Microbial and plant-assisted bioremediation of heavy metal polluted environments: A review. Int. J. Environ. Res. Public Health.

[38] Haider F.U., Liqun C., Coulter J.A., Cheema S.A., Wu J., Zhang R., Wenjun M., Farooq M. (2021). Cadmium toxicity in plants: Impacts and remediation strategies. Ecotoxicol. Environ. Saf..

[39] Grobelak A., Hiller J. (2017). Bacterial siderophores promote plant growth: Screening of catechol and hydroxamate siderophores. Int. J. Phytoremediat..

[40] Chatterjee S., Kumari S., Rath S., Priyadarshanee M., Das S. (2020). Diversity, structure and regulation of microbial metallothionein: Metal resistance and possible applications in sequestration of toxic metals. Matallomics.

[41] Rono J.K., Le Wang L., Wu X.C., Cao W.H., Zhao Y.N., Khan I.U., Yang Z.M. (2021). Identification of a new function of metallothioneinlike gene OsMT1e for cadmium detoxification and potential phytoremediation. Chemosphere.

[42] Balan B., Dhaulaniya A.S., Varma D.A. (2021). Microbial biofilm ecology, in silico study of quorum sensing receptor-ligand interactions and biofilm mediated bioremediation. Arch. Microbiol..

[43] Barra Caracciolo A., Grenni P., Garbini G.L., Rolando L., Campanale C., Aimola G., Fernandez-Lopez M., Fernandez-Gonzalez A.J., Villadas P.J., Ancona V. (2020). Characterization of the belowground microbial community in a poplar-phytoremediation strategy of a multi-contaminated soil. Front. Microb..

[44] Wyszkowska J., Boros-Lajszner E., Kucharski J. (2022). Calorific value of *Festuca rubra* biomass in the phytostabilization of soil contaminated with nickel, cobalt and cadmium which disrupt the microbiological and biochemical properties of soil. Energies.

[45] Borowik A., Wyszkowska J., Kucharski J. (2021). Microbiological study in petrol-spiked soil. Molecules.

[46] Yang Y., Dong M., Cao Y., Wang J., Tang M., Ban Y. (2017). Comparisons of soil properties, enzyme activities and microbial communities in heavy metal contaminated bulk and rhizosphere soils of *Robinia pseudoacacia* L. in the Northern foot of Qinling Mountain. Forests.

[47] Govarthanana M., Mythilib R., Selvankumarb T., Kamala-Kannanc S., Kima H. (2018). Myco-phytoremediation of arsenic- and lead-contaminated soils by *Helianthus annuus* and wood rot fungi, *Trichoderma* sp. isolated from decayed wood. Ecotoxicol. Environ. Saf..

[48] FAO (2014). World Reference Base for Soil Resources 2006.

[49] (1995). Soil Quality—Determination of Total Nitrogen—Modified Kjeldahl Method.

[50] Nelson D., Sommers L., Sparks D.L. (1996). Total carbon, organic carbon, and organic matter. Method of Soil Analysis: Chemical Methods.

[51] Egner H., Riehm H., Domingo W. (1960). Untersuchungen Über Die Chemische Bodenanalyse Als Grundlage Für Die Beurteilung Des Nährstoffzustandes Der böden. II. Chemische extractionsmethoden zur phospor- und kaliumbestimmung. Ann. Royal Agricult. Coll. Swed..

[52] Schlichting E., Blume H., Stahr K. (1995). Bodenkundliches praktikum. Pareys studientexte 81.

[53] (1998). Soil quality—Determination of cadmium, chromium, cobalt, copper, lead, manganese, nickel and zinc—Flame and electrothermal atomic absorption spectrometric methods.

[54] (2005). In Soil Quality—Determination of pH.

[55] Klute A. (1996). Methods of Soil Analysis.

[56] Bunt J.S., Rovira A.D. (1955). Microbiological studies of some subantarctic soils. J. Soil Sci..

[57] Parkinson D., Gray T.R.G., Williams S.T. (1971). Methods for Studying the Ecology of Soil Microorganisms.

[58] Martin J. (1950). Use of acid rose bengal and streptomycin in the plate method for estimating soil fungi. Soil Sci..

[59] Öhlinger R., Schinner F., Ohlinger R., Kandler E., Margesin R. (1996). Dehydrogenase activity with the substrate TTC. Methods in Soil Biology.

[60] Johnson J.L., Temple K.L. (1964). Some variables affecting the measurement of “catalase activity” in soil. Soil Sci. Soc. Am. J..

[61] Alef K., Nannipieri P. (1988). Methods in Applied Soil Microbiology and Biochemistry.

[62] Keeling J.L. (2015). The Mineralogy, Geology and Occurrences of Halloysite.

[63] Sun Y., Sun G., Xu Y., Liu W., Liang X., Wang L. (2016). Evaluation of the effectiveness of sepiolite, bentonite, and phosphate amendments on the stabilization remediation of cadmium contaminated soils. J. Environ. Manag..

[64] Hamid Y., Tang L., Hussain B., Usman M., Liu L., Ulhassan Z., He Z., Yang X. (2021). Sepiolite clay: A review of its applications to immobilize toxic metals in contaminated soils and its implications in soil–plant system. Environ. Technol. Innov..

[65] Ayaz M., Feizienė D., Tilvikienė V., Akhtar K., Stulpinaitė U., Iqbal R. (2021). Biochar role in the sustainability of agriculture and environment. Sustainability.

[66] Strachel R., Wyszkowska J., Baćmaga M. (2018). An evaluation of the effectiveness of sorbents in the remediation of soil contaminated with zinc. Water Air Soil Pollut..

[67] Boros-Lajszner E., Wyszkowska J., Kucharski J. (2018). Use of zeolite to neutralise nickel in a soil environment. Environ. Monit. Assess..

[68] Borowik A., Wyszkowska J. (2018). Remediation of soil contaminated with diesel oil. J. Elem..

[69] Boros-Lajszner E., Wyszkowska J., Kucharski J. (2020). Use of a zeolite and molecular sieve to restore homeostasis of soil contaminated with cobalt. Minerals.

[70] (2013). Microbiology of food and animal feeding stuffs—General requirements and guidance for microbiological examina-tions—Amendment 1.

[71] De Leji F.A.M., Whipps M., Lynch J.M. (1993). The use for colony development for the characterization of bacterial communities in soil and on roots. Microb. Ecol..

[72] (2017). Statistica (Data Analysis Software System). http://statistica.io.

[73] RStudio Team (2019). RStudio: Integrated Development for R.

[74] R Core Team (2019). R: A Language and Environment for Statistical Computing.

[75] Warnes G.R., Bolker B., Bonebakker L., Gentleman R., Huber W., Liaw A., Lumley T., Maechler M., Magnusson M., Moeller S. (2020). Various R Programming Tools for Plotting Data. https://CRAN.R-project.org/package=gplots.

[76] Fashola M.O., Ngole-Jeme V.M., Babalola O.O. (2016). Heavy metal pollution from gold mines: Environmental effects and bacterial strategies for resistance. Int. J. Environ. Res. Public Health.

[77] Mathivanan K., Chandirika J.U., Vinothkanna A., Yin H., Liu X., Meng D. (2021). Bacterial adaptive strategies to cope with metal toxicity in the contaminated environment—A review. Ecotoxicol. Environ. Saf..

[78] Pal C., Asiani K., Arya S., Rensing C., Stekel D.J., Larsson D.G.J., Hobman J.L. (2017). Metal resistance and its association with antibiotic resistance. Adv. Microb. Physiol..

[79] Shelton A.N., Seth E.C., Mok K.C., Han A.W., Jackson S.N., Haft D.R., Taga M.E. (2019). Uneven distribution of cobamide biosynthesis and dependence in bacteria predicted by comparative genomics. ISME J..

[80] Toledano M.B., Huang B. (2016). Microbial 2-Cys peroxiredoxins: Insights into their complex physiological roles. Mol. Cells.

[81] Kosiorek M., Wyszkowski M. (2019). Effect of cobalt on the environment and living organisms—A review. Appl. Ecol. Environ. Res..

[82] Boros-Lajszner E., Wyszkowska J., Kucharski J. (2021). Phytoremediation of soil contaminated with nickel, cadmium and cobalt. Int. J. Phytoremediat..

[83] Zaborowska M., Kucharski J., Wyszkowska J. (2016). Biological activity of soil contaminated with cobalt, tin, and molybdenum. Environ. Monit. Assess..

[84] Barras F., Fontecave M. (2011). Cobalt stress in *Escherichia coli* and *Salmonella enterica*: Molecular bases for toxicity and resistance. Metallomics.

[85] Zaborowska M., Kucharski J., Wyszkowska J. (2017). Brown algae and basalt meal in maintaining the activity of arylsulfatase of soil polluted with cadmium. Water Air Soil Pollut..

[86] Muhammad H., Wei T., Cao G., Yu S.H., Ren X.H., Jia H.L., Saleem A., Hua L., Guo J.K., Li Y. (2021). Study of soil microorganisms modified wheat straw and biochar for reducing cadmium leaching potential and bioavailability. Chemosphere.

[87] Gunina A., Kuzyakov Y. (2015). Sugars in soil and sweets for microorganisms: Review of origin, content, composition and fate. Soil Biol. Biochem..

[88] Virk A.L., Kan Z.-R., Liu B.-Y., Qi J.-Y., He C., Liu Q.-Y., Zhao X., Zhang H.-L. (2020). Impact of biochar water extract addition on soil organic carbon mineralization and C fractions in different tillage systems. Environ. Technol. Innov..

[89] Sandhu S.S., Dan U., Kumar S., Chintala R., Papiernik S.K., Malo D.D., Schumacher T.E. (2017). Analyzing the impacts of three types of biochar on soil carbon fractions and physiochemical properties in a corn-soybean rotation. Chemosphere.

[90] Steiner C., Das K.C., Garcia M., Förster B., Zech W. (2008). Charcoal and smoke extract stimulate the soil microbial community in a highly weathered xanthic Ferralsol. Pedobiologia.

[91] Ma C., Ci K., Zhu J., Sun Z., Liu Z., Li X., Zhu Y., Tang C., Wang P., Liu Z. (2021). Impacts of exogenous mineral silicon on cadmium migration and transformation in the soil-rice system and on soil health. Sci. Total Environ..

[92] Roces E., Muñiz-Menéndez M., González-Galindo J., Estaire J. (2021). Lightweight expanded clay aggregate properties based on laboratory testing. Constr. Build. Mater..

[93] Mlih R., Bydalek F., Klumpp E., Yaghi N., Bol R., Wenk J. (2020). Light-expanded clay aggregate (LECA) as a substrate in constructed wetlands—A review. Ecol. Eng..

[94] Zwikel S., Lavee H., Sarah P. (2007). Temporal dynamics in arylsulfatase enzyme activity in various microenvironments along a climatic transect in Israel. Geoderma.

[95] Wallenstein M.D., Burns R.G., Dick R.P. (2011). Ecology of extracellular enzyme activities and organic matter degradation in soil: A complex community-driven process. Methods of Soil Enzymology.

[96] Yuan B., Yue D. (2012). Soil microbial and enzymatic activities across a chronosequence of Chinese pine plantation development on the loess plateau of China. Pedosphere.

[97] Pan F., Zhang W., Liang Y., Liu S., Wang K. (2018). Increased associated effects of topography and litter and soil nutrients on soil enzyme activities and microbial biomass along vegetation successions in karst ecosystem, southwestern China. Environ. Sci. Pollut. Res..

[98] Moeskops B., Buchan D., Sleutel S., Herawaty L., Husen E., Saraswati R., Setyorini D., de Neve S. (2010). Soil microbial communities and activities under intensive organic and conventional vegetable farming in West Java, Indonesia. Appl. Soil Ecol..

[99] Qiu X., Wang J., Shi D., Li S., Zhang F., Zhang F. (2013). Syntheses, urease inhibition activities, and fluorescent properties of transition metal complexes. J. Coord. Chem..

[100] Sun Y., Zhao D., Xu Y., Wang L., Liang X., Shen Y. (2016). Effects of sepiolite on stabilization remediation of heavy metal-contaminated soil and its ecological evaluation. Front. Environ. Sci. Eng..

[101] Chao H.P., Chen S.H. (2012). Adsorption characteristics of both cationic and oxyanionic metal ions on hexadecyltrimethylammonium bromide-modified NaY zeolite. Chem. Eng. J..

[102] Jimenez-Castaneda M.E., Medina D.I. (2017). Use of surfactant modified zeolites and clays for the removal of heavy metals from water. Water.

[103] Yuan P., Tan D., Annabi-Bergaya F. (2015). Properties and applications of halloysite nanotubes: Recent research advances and future prospects. Appl. Clay Sci..

[104] Sun Y., Sun G., Xu Y., Wang L., Liang X., Lin D. (2013). Assessment of sepiolite for immobilization of cadmiumcontaminated soils. Geoderma.

[105] Abad-Valle P., Álvarez-Ayuso E., Murciego A., Pellitero E. (2016). Assessment of the use of sepiolite amendment to restore heavy metal polluted mine soil. Geoderma.

[106] Forte J., Mutiti S. (2017). Phytoremediation potential of *Helianthus annuus* and *Hydrangea paniculata* in copper and lead-contaminated soil. Water Air Soil Pollut..

[107] Rizwan M., Ali S., Rizvi H., Rinklebe J., Tsang D.C.W., Meers E., Ok Y.S., Ishaque W. (2016). Phytomanagement of heavy metals in contaminate soils using sunflower—A review. Crit. Rev. Environ. Sci. Technol..

[108] Palit S., Sharma A., Talukder G. (1994). Effects of cobalt on plants. Bot. Rev..

[109] Shahzad B., Tanveer M., Che Z., Rehman A., Cheema S.A., Sharma A., Song H., Rehman S., Zhaorong D. (2018). Role of 24-epibrassinolide (EBL) in mediating heavy metal and pesticide induced oxidative stress in plants: A review. Ecotoxicol. Environ. Saf..

[110] Bashir S., Zhu J., Fu Q., Hu H. (2018). Cadmium mobility, uptake and anti-oxidative response of water spinach (Ipomoea aquatic) under rice straw biochar, zeolite and rock phosphate as amendments. Chemosphere.

[111] Ouariti O., Gouia H., Ghorbal M.H. (1997). Responses of bean and tomato plants to cadmium—growth, mineral nutrition, and nitrate reduction. Plant Physiol. Biochem..

[112] Weryszko-Chmielewska E., Chwil M. (2005). Lead-Induced histological and ultrastructural changes in the leaves of soybean (*Glycine max* L.) Merr.). J. Plant Nutr. Soil Sci..

[113] Kim J.-Y., Oh S., Park Y.-K. (2019). Overview of biochar production from preservative treated wood with detailed analysis of biochar characteristics, heavy metal behavior, and their ecotoxicity. J. Hazard. Mater..

