# Supplementary material for: Evaluation of the Usefulness of Sorbents in the Remediation of Soil Exposed to the Pressure of Cadmium and Cobalt
Source: Materials (Basel). 2022 Aug 19;15(16):5738. doi: 10.3390/ma15165738 (PMC9415538; doi:10.3390/ma15165738)
Supplement: Supplementary file 1 [file materials-15-05738-s001.zip › materials-1812771-supplementary.pdf]

Supplementary Materials

Evaluation of the usefulness of sorbents in the remediation of soil exposed to the pressure of cadmium and cobalt

Jadwiga Wyszkowska *, Agata Borowik, Magdalena Zaborowska and Jan Kucharski

Department of Soil Science and Microbiology, University of Warmia and Mazury in Olsztyn, Plac Łódzki 3, 10-727 Olsztyn, Poland

* Correspondence: jadwiga.wyszkowska@uwm.edu.pl

Table S1. The number of organotrophic bacteria, 10^9 cfu kg^{-1} d.m. of soil

Object	C0	Cd	Co
Control	30.615^{cd}	21.002^{hi}	23.023^{f-i}
Molecular sieve	41.609^{a}	30.073^{c-e}	33.524^{c}
Halloysite	32.784^{c}	21.643^{g-i}	21.593^{g-i}
Sepiolite	33.031^{c}	19.079^{i}	18.635^{i}
Expanded clay	34.411^{bc}	32.834^{c}	24.699^{f-h}
Biochar	38.207^{ab}	22.037^{g-i}	19.819^{i}
Zeolite	32.932^{c}	27.657^{d-f}	25.685^{e-g}

C0 – uncontaminated soil, Cd – cadmium ion, Co – cobalt ion. Homogeneous groups denoted with letters (a-i).

Table S2. The number of actinomycetes, 10^9 cfu kg^{-1} d.m. of soil

Object	C0	Cd	Co
Control	16.170^{g-j}	15.875^{h-k}	13.410^{j-l}
Molecular sieve	17.354^{e-h}	29.136^{a}	17.008^{f-i}
Halloysite	20.410^{c-e}	12.966^{kl}	10.599^{l}
Sepiolite	20.854^{cd}	19.276^{d-f}	13.459^{j-l}
Expanded clay	22.530^{bc}	17.501^{e-h}	19.720^{c-f}
Biochar	19.276^{d-f}	25.340^{b}	15.677^{h-k}
Zeolite	14.100^{i-k}	19.128^{d-g}	16.170^{g-j}

C0 – uncontaminated soil, Cd – cadmium ion, Co – cobalt ion. Homogeneous groups denoted with letters (a-l).

Table S3. The number of fungi, 10^7 cfu kg^{-1} d.m. of soil

Object	C0	Cd	Co
Control	6.754^{d-h}	5.522^{h-k}	4.388^{i}
Molecular sieve	7.592^{d-f}	6.508^{e-i}	5.078^{jk}
Halloysite	9.712^{ab}	7.494^{def}	5.374^{i-k}
Sepiolite	7.099^{d-g}	6.064^{g-j}	5.669^{h-k}
Expanded clay	10.304^{ab}	7.937^{cd}	7.789^{c-e}
Biochar	10.599^{a}	7.592^{d-f}	6.409^{f-j}
Zeolite	8.973^{bc}	7.148^{d-g}	5.423^{h-k}

C0 – uncontaminated soil, Cd – cadmium ion, Co – cobalt ion. Homogeneous groups denoted with letters (a-k).

Table S4. Dehydrogenases activity, μmol TPF · h^{-1} · kg^{-1} d.m. of soil

Object	C0	Cd	Co
Control	6.231^{c}	1.002^{g-i}	0.357^{i}
Molecular sieve	9.134^{a}	3.243^{d}	2.971^{de}
Halloysite	6.502^{c}	1.969^{fg}	1.358^{gh}
Sepiolite	6.564^{c}	1.154^{g-i}	0.934^{hi}
Expanded clay	7.776^{b}	1.698^{f-h}	0.900^{hi}
Biochar	6.927^{bc}	2.343^{ef}	2.037^{fg}
Zeolite	9.694^{a}	1.222^{gh}	3.226^{de}

C0 – uncontaminated soil, Cd – cadmium ion, Co – cobalt ion. Homogeneous groups denoted with letters (a-i).

Table S5. Catalase activity, mol O_2 h^{-1} kg^{-1} d.m. of soil

Object	C0	Cd	Co
Control	0.338^{d-f}	0.293^{k-m}	0.278^{m}
Molecular sieve	0.357^{bc}	0.327^{e-h}	0.315^{hij}
Halloysite	0.364^{ab}	0.345^{cd}	0.319^{g-i}
Sepiolite	0.349^{b-d}	0.342^{c-e}	0.286^{lm}
Expanded clay	0.342^{c-e}	0.334^{d-g}	0.293^{k-m}
Biochar	0.379^{a}	0.323^{f-h}	0.304^{i-k}
Zeolite	0.375^{a}	0.342^{c-e}	0.301^{j-l}

C0 – uncontaminated soil, Cd – cadmium ion, Co – cobalt ion. Homogeneous groups denoted with letters (a-m).

Table S6. Urease activity, mmol $N\text{-}NH_4$ h^{-1} kg^{-1} d.m. of soil

Object	C0	Cd	Co
Control	0.223^{c}	0.146^{f}	0.116^{g}
Molecular sieve	0.254^{ab}	0.162^{d-f}	0.154^{ef}
Halloysite	0.239^{bc}	0.216^{c}	0.139^{fg}
Sepiolite	0.239^{bc}	0.185^{d}	0.162^{d-f}
Expanded clay	0.254^{ab}	0.177^{de}	0.146^{f}
Biochar	0.262^{ab}	0.177^{de}	0.154^{ef}
Zeolite	0.277^{a}	0.185^{d}	0.162^{d-f}

C0 – uncontaminated soil, Cd – cadmium ion, Co – cobalt ion. Homogeneous groups denoted with letters (a-g).

Table S7. Alkaline phosphatase activity, mmol PNP kg^{-1} h^{-1} kg^{-1} d.m. of soil

Object	C0	Cd	Co
Control	1.349^{cd}	0.568^{k}	0.814^{h}
Molecular sieve	1.406^{bc}	0.682^{i}	1.306^{d}
Halloysite	1.404^{bc}	0.581^{jk}	0.874^{gh}
Sepiolite	1.544^{a}	0.649^{ij}	1.049^{e}
Expanded clay	1.424^{b}	0.574^{k}	0.909^{g}
Biochar	1.574^{a}	0.589^{jk}	1.117^{e}
Zeolite	1.578^{a}	0.644^{ij}	0.980^{f}

C0 – uncontaminated soil, Cd – cadmium ion, Co – cobalt ion. Homogeneous groups denoted with letters (a-k).

Table S8. Acid phosphatase activity, mmol PNP h^{-1} kg^{-1} d.m. of soil

Object	C0	Cd	Co
Control	1.275^{de}	0.812^{h}	0.897^{h}
Molecular sieve	1.593^{b}	1.312^{d}	1.106^{fg}
Halloysite	1.483^{c}	1.124^{fg}	1.086^{g}
Sepiolite	1.755^{a}	1.208^{ef}	1.129^{fg}
Expanded clay	1.533^{bc}	1.055^{g}	1.074^{g}
Biochar	1.567^{bc}	1.050^{g}	1.036^{g}
Zeolite	1.599^{b}	1.038^{g}	1.078^{g}

C0 – uncontaminated soil, Cd – cadmium ion, Co – cobalt ion. Homogeneous groups denoted with letters (a-h).

Table S9. Arylsulfatase activity, mmol PNS h^{-1} kg^{-1} d.m. of soil

Object	C0	Cd	Co
Control	0.188^{bc}	0.125^{i}	$0.144^{f\text{-}i}$
Molecular sieve	0.218^{a}	$0.156^{d\text{-}h}$	$0.170^{c\text{-}e}$
Halloysite	0.216^{a}	$0.140^{g\text{-}i}$	$0.162^{d\text{-}g}$
Sepiolite	0.204^{ab}	0.132^{hi}	$0.168^{c\text{-}f}$
Expanded clay	0.200^{ab}	$0.144^{f\text{-}i}$	$0.166^{c\text{-}f}$
Biochar	0.202^{ab}	$0.148^{e\text{-}i}$	$0.170^{c\text{-}e}$
Zeolite	0.221^{a}	0.131^{i}	$0.180^{b\text{-}d}$

C0 – uncontaminated soil, Cd – cadmium ion, Co – cobalt ion. Homogeneous groups denoted with letters (a-i).

Table S10. β-glucosidase activity, mmol PNG h^{-1} kg^{-1} d.m. of soil

Object	C0	Cd	Co
Control	0.550^{hi}	0.444^{j}	0.498^{ij}
Molecular sieve	$0.661^{c\text{-}f}$	$0.629^{e\text{-}g}$	$0.641^{d\text{-}g}$
Halloysite	0.761^{a}	$0.663^{c\text{-}f}$	0.751^{ab}
Sepiolite	$0.709^{a\text{-}d}$	$0.613^{f\text{-}h}$	0.583^{gh}
Expanded clay	$0.665^{c\text{-}f}$	$0.608^{f\text{-}h}$	$0.604^{f\text{-}h}$
Biochar	$0.702^{a\text{-}e}$	$0.664^{c\text{-}f}$	$0.660^{c\text{-}g}$
Zeolite	$0.721^{a\text{-}c}$	$0.609^{f\text{-}h}$	$0.681^{b\text{-}f}$

C0 – uncontaminated soil, Cd – cadmium ion, Co – cobalt ion. Homogeneous groups denoted with letters (a-j).

Table S11. Yield of aboveground parts of sunflower (*Helianthus annuus*), g d.m. on pot^{-1}

Object	C0	Cd	Co
Control	25.27^{b}	$10.34^{f\text{-}i}$	6.78^{i}
Molecular sieve	27.26^{ab}	18.63^{c}	$13.11^{d\text{-}f}$
Halloysite	27.66^{ab}	$13.00^{d\text{-}f}$	$9.12^{g\text{-}i}$
Sepiolite	27.04^{ab}	16.40^{cd}	8.03^{hi}
Expanded clay	26.36^{ab}	14.43^{de}	7.99^{hi}
Biochar	29.89^{a}	$12.18^{e\text{-}g}$	$8.67^{g\text{-}i}$
Zeolite	29.06^{a}	$11.34^{e\text{-}h}$	$10.19^{f\text{-}i}$

C0 – uncontaminated soil, Cd – cadmium ion, Co – cobalt ion. Homogeneous groups denoted with letters (a-i).

Table S12. Sunflower (*Helianthus annuus*) root yield, g d.m. on pot^{-1}

Object	C0	Cd	Co
Control	5.18^{c}	1.19^{f}	1.56^{d-f}
Molecular sieve	5.89^{c}	2.18^{d-f}	2.53^{d}
Halloysite	7.63^{a}	1.40^{ef}	1.89^{d-f}
Sepiolite	6.06^{bc}	2.24^{d-f}	2.32^{de}
Expanded clay	5.63^{c}	2.07^{d-f}	1.73^{d-f}
Biochar	5.80^{c}	1.72^{d-f}	2.04^{d-f}
Zeolite	7.15^{ab}	1.63^{d-f}	2.19^{d-f}

C0 – uncontaminated soil, Cd – cadmium ion, Co – cobalt ion. Homogeneous groups denoted with letters (a-f).
